# Effectiveness and cost-effectiveness of humanistic counselling in schools for young people with emotional distress (ETHOS): study protocol for a randomised controlled trial

**DOI:** 10.1186/s13063-018-2538-2

**Published:** 2018-03-09

**Authors:** Megan Rose Stafford, Mick Cooper, Michael Barkham, Jeni Beecham, Peter Bower, Karen Cromarty, Andrew J. B. Fugard, Charlie Jackson, Peter Pearce, Rebekah Ryder, Cathy Street

**Affiliations:** 1 Department of Psychology, University of Roehampton, Whitelands College, Holybourne Avenue, London, SW15 4JD UK; 2 Centre for Psychological Services Research, Department of Psychology, University of Sheffield, Sheffield, S10 2TN UK; 3 Personal Social Services Research Unit (PSSRU) at the London School of Economics, Houghton St, London, WC2A 2AE UK; 4 PSSRU at the Faculty of Social Sciences, Cornwallis North East, University of Kent, Canterbury, Kent CT2 7NF UK; 5 NIHR School for Primary Care Research, Division of Population Health, Health Services Research and Primary Care, Manchester Academic Health Science Centre, University of Manchester, Williamson Building, Oxford Road, Manchester, M13 9PL UK; 6 Karen Cromarty Consultancy, 4 Bamburgh Road, Durham, DH1 5NW UK; 7 Research Department of Clinical, Educational and Health Psychology, University College London, Gower Street, London, WC1E 6BT UK; 8 Department of Psychosocial Studies, Birkbeck, University of London, 30 Russell Square, London, WC1B 5DT UK; 9 BACP House, 15 St John’s Business Park, Lutterworth, Leicester, LE17 4HB UK; 10 Applied Social and Organisational Sciences, Metanoia Institute, 13 Gunnersbury Ave, Ealing, London, W5 3XD UK; 11 Formerly the National Children’s Bureau, Research and Policy, 8 Wakley Street, London, EC1V 7QE UK

**Keywords:** School-based humanistic counselling, Randomised controlled trial, Young people, Psychological distress, Cost-effectiveness

## Abstract

**Background:**

One in ten children in Britain have been identified as experiencing a diagnosable mental health disorder. School-based humanistic counselling (SBHC) may help young people identify, address, and overcome psychological distress. Data from four pilot trials suggest that SBHC may be clinically effective. However, a fully powered randomised controlled trial (RCT) is needed to provide a robust test of its effectiveness, to assess its cost-effectiveness, and to determine the process of change.

**Methods/design:**

The Effectiveness and Cost-effectiveness Trial of Humanistic Counselling in Schools (ETHOS) is a two-arm, parallel-group RCT comparing the clinical and cost-effectiveness of SBHC with Pastoral Care as Usual (PCAU) in school settings. Eligibility criteria for young people include being between 13 and 16 years of age and experiencing moderate to severe levels of emotional distress. Participants are randomised to receive either SBHC or PCAU. SBHC is delivered in up to 10 weekly, individual sessions in their school with a qualified, experienced counsellor who has also received training using a clinical practice manual. Adherence to the SBHC model is assessed by a sub-team of auditors and in clinical supervision. PCAU consists of the schools’ pre-existing systems for supporting the emotional health and well-being of students. The primary outcomes are psychological distress measured using the Young Person’s Clinical Outcomes in Routine Evaluation (YP-CORE) and costs evaluated using the Client Service Receipt Inventory (CSRI). Secondary outcomes include psychological difficulties, levels of depression, anxiety and self-esteem, well-being, school engagement, educational outcomes and achievement of personal goals. Qualitative interviews with participants, parents and school staff will look to identify the mechanisms of change in SBHC. Researchers administering the measures are blind to allocation. The trial requires *n* = 306 participants (*n* = 153 in each group), with 90% power to detect a standardised mean difference (SMD) of 0.5. An intention-to-treat analysis will be undertaken.

**Discussion:**

This RCT is powered to detect clinically meaningful differences, and will make a major contribution to the evidence base for mental health provision for adolescents. It will have implications for all stakeholders, including policy-makers, statutory advisory bodies for child welfare, head teachers, children and young people practitioners, child welfare and parenting organisations, and young people.

**Trial registration:**

Controlled Trials International Standard Randomised Controlled Trial Number (ISRCTN) Registry, ID: ISRCTN10460622. Registered on 11 May 2016.

**Electronic supplementary material:**

The online version of this article (10.1186/s13063-018-2538-2) contains supplementary material, which is available to authorized users.

## Background

One in ten children in Britain have been identified as experiencing a diagnosable mental health disorder [1]. A study conducted by The Prince’s Trust [2] reported that 30% of young people aged between 16 and 25 years reported ‘always’ or ‘often’ feeling ‘down’ or ‘depressed’, and 21% felt that they did not receive the support they needed from school. A number of longitudinal studies have highlighted that untreated mental health and behavioural problems in childhood can have profound longstanding, social and economic consequences in adulthood, including increased contact with the criminal justice system and reduced levels of employment [3–6]. Britain may be falling behind in promoting well-being in children [7], where high levels of distress, low self-esteem, and self-harm are seen in this age group [8]. There is some evidence that levels of mental health problems in children and young people are increasing [9]. Research has indicated that children with persistent behavioural or emotional difficulties are more likely to be excluded from school, and more likely to leave schools without obtaining their educational qualifications [1, 10], whereas emotional, behavioural, social and school well-being predict higher levels of academic achievement and engagement in school [11].

Evidence suggests that targeted school-based interventions lead to improvements in well-being and mental health, yielding reduced levels of exclusion by 31% and improved pupil attainment [12]. As childhood behavioural and emotional difficulties often continue into adulthood (e.g. [13, 14]), investing in support for young people with behavioural and emotional difficulties can help them both achieve academically, and may also improve longer-term outcomes such as employment and health. Knapp *et al.* [15] demonstrated that interventions targeted towards children and young people lead to savings for the public sector, particularly the NHS and education.

### Summary of the existing academic literature

Meta-analysis of psychotherapeutic interventions with children and adolescents indicate that they are effective, with effect sizes comparing treatment to no treatment hovering in the 0.70 range [16, 17]. An effect size of 0.45 (95% confidence interval (CI) [0.37–0.53]) has been found for school-based interventions specifically [18]. Evidence primarily comes from trials of cognitive behaviour therapy (CBT), which is an established intervention for clinical presentations such as anxiety and depression in children and young people (e.g. National Institute for Health and Care Excellence [19]). However, many young people referred to school counselling services do not present with specific clinical disorders [20]. Rather, they are more likely to be experiencing psychological distress as a result of a range of life difficulties such as family issues, bullying, or academic problems.

There is a need for school-based interventions that can help young people identify, address, and overcome, the distress that arises from these life challenges; and that can minimise the likelihood of this distress developing into more severe psychological problems in adulthood. One potential intervention that may achieve this is school-based counselling [21, 22]. In the UK, school-based counselling provision tends to be based around a humanistic, person-centred model of practice [16, 23] with a focus on young people’s emotional difficulties and on a one-to-one basis with a counsellor [24]. Within the UK, the research to date suggests that it is perceived by children and pastoral care staff as a highly accessible, non-stigmatising, and effective form of early intervention for reducing psychological distress [20]. It has also been associated with positive change [20, 25]. For example, Cooper [20] and Cooper *et al.* [25] found significant reductions in psychological distress pre- and post-counselling (weighted mean difference (WMD) = 0.81, 95% CI [0.76–0.86]; and *d* = 1.49, 95% CI [1.29–1.69], respectively). Furthermore, secondary school pupils have reported that attending school-based counselling services positively impacted on their studying and learning [26]. School management has reported perceived improvements in attainment, attendance, and behaviour of young people who have accessed school-based counselling services [27]. Similarly, McElearney *et al.* [28] reported that school-based counselling interventions in Northern Ireland were effective for pupils who have been bullied. However, school-based counselling in the UK is heterogeneous, and with limited evidence of effectiveness.

In 2009, ‘school-based humanistic counselling’ (SBHC) was developed as a standardised form of school-based counselling [24]. The humanistic orientation of this approach reflects the predominantly person-centred/humanistic style of British school-based counsellors [20, 22]. However, it is based in evidence-based competences for humanistic therapies [29]. A competency framework, which forms a more extended basis for SBHC, has now been developed for those delivering humanistic counselling to 11–18 year-olds [30].

### Pilot studies

Four pilot studies of manualised SBHC against Pastoral Care As Usual (PCAU) (with the offer of counselling once they had completed participation in the trial), for young people experiencing moderate to severe levels of emotional distress have been conducted [24, 31–33]. The first pilot study assessed the feasibility of conducting a trial of this nature, including likely recruitment and follow-up rates, and trial procedures [24]. The studies that followed aimed to contribute further data estimating the effectiveness of SBHC, and improve trial procedures including extending the intervention period, adding a 6-month follow-up, post-intervention time point, and aiming to include a more ethnically diverse sample [31–33]. In three of these studies recruitment was through pastoral care referral, that is to say, via an already established team of professionals within the school tasked with supporting the emotional health and well-being of the school’s pupils [31–33]. The total number of participants in each pilot study ranged from 32 to 64 and SBHC was delivered weekly for up to 10 weeks. In terms of feasibility, these studies found that recruitment levels were acceptable, with an average of 10.1 pupils recruited per school per academic year. Completion rates were also acceptable, with 76.2% to 100% of pupils completing the study to endpoint. All procedures were acceptable to the schools and young people involved, with no ethical concerns raised.

A pooled analysis of data across these four pilot studies suggests that SBHC brings about statistically significant, medium to large reductions in psychological distress as compared to PCAU, up to 12 weeks from assessment. On the primary outcome in each pilot study, the Young Person’s Clinical Outcomes in Routine Evaluation (YP-CORE) [34] the mean effect size (Hedges’ *g*) for counselling compared to PCAU at mid-point (6 weeks from baseline) was 0.47 (95% CI [0.09 to 0.88]; counselling *n* = 58, control *n* = 60) and at endpoint (3 months from baseline) it was 0.72 (95% CI [0.34 to 1.10], counselling *n* = 63, control *n* = 63). The mean effect size was not statistically significant at 6 months (*g* = 0.44, 95% CI [− 0.17 to 1.06]; counselling *n* = 23, control *n* = 23) or at 9 months from baseline (*g* = − 0.16, 95% CI [− 0.73 to 0.47]; counselling *n* = 21, control *n* = 24). However, these sample sizes were small.

In addition, the preliminary findings of Pearce *et al.* [33] suggest that in the short-term, counselling compared to school-based pastoral care, primary and hospital care, and community-based services, had the greatest impact on within-school costs, reducing the amount of time/costs form teachers and pastoral care staff spent with these pupils. These initial results indicate that counselling may be a more appropriate and more effective resource.

### Rationale for the current study

These pilot studies indicate that a trial of SBHC is feasible and that there are initial indications of a short-term effect. However, a trial that is powered to detect clinically meaningful differences is required which can provide more comprehensive data on the effectiveness of SBHC, in particular its longer-term effects, cost-effectiveness and impact on educational outcomes; as well as identifying mechanisms of change.

Accordingly, this paper sets out the protocol for a randomised controlled trial (RCT) in a school setting, to determine the clinical and cost-effectiveness of SBHC compared to PCAU. The protocol adheres to the Standard Protocol Items: Recommendations for Intervention Trials (SPIRIT) Checklist which is available as an additional file (Additional file 1).

## Aims

The primary objectives are to evaluate the clinical and cost-effectiveness of SBHC in reducing psychological distress in young people, as compared with PCAU. The secondary aims are to evaluate the effectiveness of SBHC as compared to PCAU on a range of additional outcomes, including psychological difficulties, symptoms of depression and generalised anxiety, self-esteem, personal goals, well-being, and educational engagement. In addition we aim to identify the mechanisms of change in SBHC.

## Methods/design

The ETHOS study is a two-arm, parallel-group RCT comparing the clinical and cost-effectiveness of SBHC with PCAU in a school setting. PCAU is used as a comparison group, rather than an alternative active treatment (e.g. CBT) so that we are able to examine the effects of SBHC against standard (non-counselling) provision in schools in the UK (for further details regarding SBHC and PCAU, see below).

### Participants: eligibility criteria

Participants are young people attending one of 18 secondary schools across London who meet the inclusion criteria, which includes being between 13 and 16 years of age at the time of assessment and experiencing moderate to severe levels of psychological distress as assessed by a score of ≥ 5 on the Strengths and Difficulties Questionnaire Emotional Symptoms (SDQ-ES) Scale [35] (see below, ‘Assessment’). In addition, young people need to be considered capable of comprehending the outcome measurement forms, with a guide English reading age of 13 years, want to participate in counselling and not be in current receipt of counselling or any other therapeutic intervention that may be impeded through participation in the trial. In order to increase the likelihood of participant attendance at research meetings, we ask that all potential participants have a school attendance record of at least 85% as assessed by the school. Young people are excluded from participation if they are unable to provide informed consent (not ‘Gillick competent’), their parent/carer has not provided informed consent, or the young person is planning to leave the school within the academic year. Additionally, the young person is not included in the trial if they are deemed at risk of serious harm to self or others at the time of assessment. We ask all young people if they are, in principle, willing to complete all outcomes measures at each research meeting, and for their counselling sessions to be audio-recorded (on the understanding that they may exercise their right to request that the recording device be turned off at any point during a session).

### Study setting

The study is being conducted in 18 schools across London, UK. All participating schools are located in urban areas, with 17 (94%) categorised as ‘major urban’ [36]. Six schools (33%) are located within the areas considered ‘most deprived’, and two (11%) schools in ‘least deprived’ areas of England (using the Index of Multiple Deprivation (IMD)) [37]. Over half (56%) of included schools fall between these two categories. The percentage of pupils eligible for free school meals, across included schools, ranges from 11 to 53% (median 33%), with over 25% of pupils eligible for free schools meals in 11 (61%) included schools. Nine (50%) schools have a pupil population of between 700 and 1100 students, five schools’ (28%) population of pupils exceeds 1100, and three (17%) schools are smaller than 700 students. Five (28%) schools are single-sex schools, with three of these single-sex girls’ schools (17% of all included schools). Schools that already had counselling provision, or planned to incorporate counselling provision during the trial period; or whose first language was not English, have not been included in the trial.

### Intervention: School-based humanistic counselling (SBHC)

SBHC is based on competences for humanistic counselling with young people aged 11–18 years [30] and follows a clinical practice manual developed for the trial (Kirkbride, 2016, unpublished manuscript). It is based on the theory that distressed young people have the capacity to successfully address their difficulties if they can talk them through with an empathic, supportive, and qualified counsellor. School-based humanistic counsellors use a range of techniques to facilitate this process, including active listening, empathic reflections, inviting young people to access and express underlying emotions and needs, and helping clients to reflect on, and make sense of, their experiences and behaviours. Young people are also encouraged to consider the range of options that they are facing, and to make choices that are most likely to be helpful within their given circumstances. As part of the intervention, young people participating in the trial are asked to complete a sessional outcome measure in accordance with the recommendations outlined in the Children and Young People Practice Research Network (CYP PRN) report that highlights the clinical utility of employing a regular feedback tool in counselling [38]. Increasingly, the use of such a tool is becoming standard practice within the field and within Children and Young People’s Increasing Access to Psychological Therapies services (CYP IAPT). Our sessional measure is the Outcomes Rating Scale (ORS) [39] which assesses the following areas of life functioning: personal well-being, family and close relationships, social relationships and general well-being; and is integrated into the therapeutic dialogue.

#### Delivery of SBHC

SBHC is delivered over ten school weeks, in up to ten weekly, face-to-face sessions of between 45 and 60 min each (depending the length of a school period) on an individual basis. All participants allocated to the SBHC group are able to continue accessing their school’s pastoral care services as needed. There are no modifications to the trial intervention, such as an extension to the agreed number of sessions. Participants may choose to end their counselling sessions before they have completed ten sessions in total.

#### SBHC counsellors

SBHC is delivered by counsellors who have completed at a minimum, a professional, diploma-level training in humanistic, person-centred or humanistic-integrative counselling, and accumulated a minimum of 450 h of counselling/psychotherapy practice covered by at least 1.5 h of supervision per month. All counsellors are members of the British Association for Counselling and Psychotherapy (BACP) or equivalent, and abide by the BACP’s Ethical Framework for the Counselling Professions [40].

#### Training in SBHC

Prior to the trial commencing, SBHC clinical practice training and ETHOS protocol training are provided to all counsellors. Training is designed to build on counsellors’ prior experience of working therapeutically with young people, using a humanistic model. Training consists of a 5-day taught programme, using the clinical practice manual developed for the trial, with 1 day devoted to the trial protocols. In addition, supervisors are employed to oversee the delivery of SBHC by the trial counsellors, in line with best practice guidelines for frequency and amount, as set out by BACP. Supervisors are provided with a 2-day training programme using a manual for supervision practice developed for the trial.

### Control group: Pastoral Care as Usual (PCAU)

‘Pastoral care’ in UK schools is a generic, umbrella term used to describe targeted services offered by the school to support the well-being of pupils on roll. Such services vary across schools and while there is no one approach, the existence of such services within schools is a standard practice and seen as inextricably linked to teaching, learning, and the curriculum. For many UK schools, in particular those with limited financial resources, pastoral care is the only provision on offer to accommodate pupils’ well-being needs as targeted interventions, such as counselling, are seen as too costly. Despite the differences in pastoral care services across UK schools, there are also some similarities in the models and approaches employed. For example, form tutors may be tasked with monitoring any emotional difficulties as they arise for pupils in their form group, policies and guidance will be in place relating to bullying and behaviour, schools will have established links to local external services or agencies, for example Child and Adolescent Mental Health Services (CAMHS). School pastoral care services in the UK do not routinely offer counselling, and differ from humanistic counselling in several ways: (1) pastoral care is offered by any member of staff, whatever their qualifications, (2) pastoral care is advice-giving and directive, (3) pastoral care does not involve a collaborative contract, with an agreement on boundaries, confidentiality, or therapeutic goals, (4) pastoral care is most often provided ‘ad hoc’, and is not time limited while the pupil is on roll.

PCAU within the current study, therefore, consists of the included schools’ pre-existing, ‘as usual’ systems for supporting the emotional health and well-being of young people within their particular school, which may involve a personal tutor, or a school inclusion lead meeting regularly with the young person to speak about their difficulties. PCAU does not consist of humanistic counselling, but additional school and non-school-based interventions for young people may be provided, such as a short placement in a learning support unit, some one-to-one support or group work from a school nurse, learning mentor, educational psychologist or behaviour support team, and interagency meetings (for example, the creation of a Common Assessment Framework). The intensity and length of support will depend upon the specific needs of each young person. The number of interventions and the specific form of support is recorded by the pastoral care team in a pastoral care log (see below). Participants randomly assigned to the PCAU condition will be offered the opportunity to access SBHC 6 to 9 months after their assessment.

### SBHC adherence monitoring

Adherence to the SBHC model is assessed at two levels: (1) counsellor level and (2) client level.

Counsellor-level assessment uses a young-person-adapted version of the Person Centred and Experiential Psychotherapy Rating Scale [41], the PCEPS-YP, developed specifically for this trial. ‘Calibration tapes’ have been developed, using a sample of recordings which have been rated by national experts, using the PCEPS-YP. Calibration tapes provide a target rating for use in the training and standardisation of subsequent ratings. The national experts for SBHC are based at the Metanoia Institute, Edge Hill University, the University of Nottingham, and the University of Strathclyde.

An independent sub-team of auditors will then rate 20-min recorded segments of counselling sessions which will be selected at random. All counsellors will be sampled. For each counsellor, auditors will assess sessions from four clients, spread across the duration of the counsellor’s involvement in the trial. This will include two recordings per client (and. therefore eight recordings in total, per counsellor). The 20-min segments will be randomly selected from one session in the first half of the counselling work with each selected client (excluding the first session), and one session from the second half of their work with that client (excluding the last session). This sampling strategy will yield a total of 144 recordings to be rated in the trial.

At the client level, adherence to SBHC competences are audited in supervision using a short version of the PCEPS, which has been adapted for work with young people (PCEPS-YP-S). To audit sessions, supervisor and counsellor listen to a segment of a minimum of 10 min in length together, per client, with each of them completing, comparing and discussing their ratings.

Adherence of supervision of SBHC practice will be monitored using an ETHOS supervision adherence form developed for the trial. An independent sub-team of auditors will rate 20-min recorded segments of supervision sessions which will be selected at random. Every counsellor, working with each supervisor, will be sampled. Auditors will assess two sessions for each counsellor the supervisor works with, spread across the duration of the counsellor’s involvement in the trial (and, therefore, 36 recordings will be assessed in total).

### Consent process

School pastoral care teams are the initial point of contact for entry into the trial. Prior to any assessments of eligibility, pastoral care staff, briefed and trained on the details of the trial, identify which young people in their school they deem to be potentially eligible using the trial eligibility criteria. Pastoral care staff approach, and invite, potentially eligible young people for a pre-screening meeting to discuss the project, and if the young person expresses an interest in participating in the trial, and is willing to ask their parents/carers for signed consent, the pastoral care teacher sends a standardised trial letter to their parents/carers with information about the trial and consenting procedures. This method of identifying young people who may benefit from counselling reflects a relatively commonplace procedure in pastoral care referral to counselling services in UK schools. At this stage, it is explained to parents/carers that not every young person who is put forward to take part in the trial is eligible, and that assessment procedures are not a judgement about the young person or their problems, but an assessment of trial eligibility. Once parents/carers ‘opt in’ informed consent is obtained pastoral care staff make an initial referral to the ETHOS research team, on the basis of likelihood that the young person they have identified will meet all eligibility criteria during the assessment meeting. Informed parent/carer consent is obtained either in writing, or via the telephone with a member of the pastoral care staff or an ETHOS researcher acting as a proxy to obtain consent in this way. Consent obtained by proxy is either audio-recorded, or witnessed by a third party.

On receiving parent/carer consent, the young person is then invited to an assessment meeting with an ETHOS assessor. Assessment meetings are held in a confidential and secure environment within the school. The assessor talks the young person through the aims and nature of the trial, and explains to them the assessment and intervention procedures using an Information Sheet for Young People developed for the trial, during which time the young person has the opportunity to ask any questions they have about their potential involvement. During this meeting, the assessor makes a judgement as to whether they believe that the young person is of sufficient maturity and understanding to consent to participate in the research study, using Competence to Consent Guidelines developed for the trial. This judgement is made prior to any other assessment of eligibility; and the young person’s written, informed consent is obtained prior to any study procedures being carried out, or outcome measures being administered. The young person’s written, informed consent is required in addition to their parent’s/carer’s.

### Assessment

**Fig. 1:**
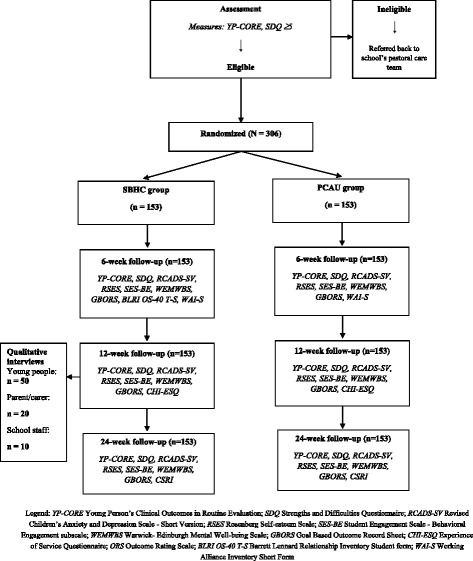
Study flow chart of referral, screening and allocation of participants to the ETHOS study

Where consent has been obtained from both the young person and their parent/carer, each young person is assessed for their eligibility into the trial by an assessor. All assessors are provided with comprehensive training in the study and the purposes and principles of the assessments. The primary measure for determining suitability for the trial is the Strength and Difficulties Questionnaire (SDQ) [35]. The SDQ measures psychological difficulties along 25 attributes, divided equally between five sub-scales: emotional symptoms, conduct problems, hyperactivity/inattention, peer-relationship problems, and prosocial behaviour. Respondents are required to respond to each of the 25 attributes by reflecting on the last 6 months. For each sub-scale, the score ranges from 0 to 10, with higher scores indicating greater difficulties. Young people completing the SDQ at the assessment meeting for the current trial are required to score ≥ 5 on the emotional symptoms sub-scale (SDQ-ES). Risk assessment is carried out following a discussion with the young person, and using the YP-CORE item 4 (‘I’ve thought of hurting myself”) which has a score range of 0 to 4, with higher scores indicating greater psychological distress [34]. If a young person scores ≥ 1 (on YP-CORE item 4) the assessor asks, in a sensitive and gentle manner, whether they feel that they are at risk to themselves or others, and makes a professional judgement using guidelines for assessing and managing risk developed for the trial. It is explained to the young person that, as there is an equal chance of being allocated to PCAU for 6 to 9 months prior to counselling, it is important for the research and pastoral care teams to know whether they need more immediate attention. If the young person is assessed as being in immediate danger or at risk of serious harm, procedures for onward disclosure and referral are followed. Assessors review the remaining criteria for participating in the trial with the young person before confirming their eligibility. All young people are asked to complete a demographic questionnaire in the first part of the assessment meeting. Figure 1 presents a flow chart of the progress of participants through the trial.

### Randomisation

Consenting young people are allocated to one of two groups, SBHC or PCAU, via remote access to the central randomisation procedure that is hosted by an in-house vb.net application with a Standard Query Language (SQL) server backend at the Clinical Trials Unit, Manchester Academic Health Science Centre, University of Manchester (MAHSC-CTU). This is restricted access and users other than the system owner do not have access to future allocations. Sequence generation is, therefore, concealed from both the assessor and young person, as well as from the rest of the core research team. The randomisation ratio is 1:1 using the method of permuted blocks within school strata with adjacent block sizes themselves varying randomly within pre-specified limits.

### Blinding

Allocation of each participant to SBHC or PCAU is recorded in a separate Assessment Log used only by the assessors. Blinding of participants, providers and assessors is not possible, so this trial employs a ‘tester blind’ design wherein testers are blind to the young person’s allocation for the duration of the trial. To help ensure the success of the blind, a different tester is employed at 6 weeks/mid-point, 12 weeks/endpoint and 24 weeks/follow-up for each participant. The success of the blind is assessed by asking testers to report whether the participant or member of the pastoral care staff have revealed what group the participant is allocated to, on the predesigned Case Report Form (CRF) developed for the trial. Participants and school staff are asked not to reveal the group to which participants have been assigned, as far as is possible.

It is necessary to unmask the allocation of participants to testers conducting the mid-point/6-week meeting to allow the administration of the Working Alliance Inventory Short Form (WAI-S) [42] in the SBHC group (see below, ‘Measures’). Therefore, three separate CRFs have been developed at the mid-point/6-week meeting to ensure that the remaining measures are administered under blind conditions: CRF 1 contains all measures for administration to both groups and is used in the first part of the meeting, CRFs 2 and 3 contain measures only applicable to the SBHC or PCAU group, respectively, and are used in the second part of the meeting. Once CRF 1 has been completed by the participant, the participant’s allocation is revealed to the tester who opens a sealed envelope containing information as to which group the participant is in, and which CRF (2 or 3) should, therefore, be administered. Only testers conducting the mid-point/6-week test have access to the contents of the envelope, which is destroyed following use. The only measures completed following the unmasking of the tester are those relevant to the participant’s specific allocation.

### Measures

**Fig. 2:**
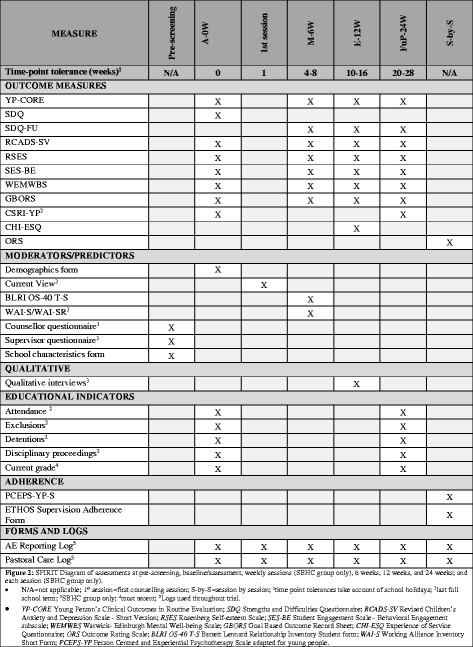
Standard Protocol Item: Recommendations for Interventional Trials (SPIRIT) diagram of assessments at pre-screening, baseline/assessment, first session, session by session, mid-point/6 weeks, endpoint/12 weeks and follow-up/24 weeks

Once eligibility has been established, the second part of the assessment meeting requires participants to complete a battery of measures. In addition, eligible participants attend research meetings at 6 (mid-point), 12 (endpoint) and 24 (follow-up) weeks following their assessment meeting. Pastoral care staff at each included school work with the core research team to schedule research meetings with participants, and follow-up with participants if they are absent (e.g. due to sickness or a school trip). Research meetings are scheduled to fit into the each school’s individual daily timetable. A summary of the assessments, per time point, are presented in Fig. 2 and described below.

#### Primary outcomes

##### Young Person’s Clinical Outcomes in Routine Evaluation (YP-CORE)

The primary outcome measure is the YP-CORE [34], a 10-item, self-report, 5-point Likert-type scale measuring psychological distress. Participants are asked to rate how they have been feeling over the last week (prior to completing the questionnaire) in relation to 10 items. Individual item scores range from 0 (‘not at all’) to 4 (‘most or all of the time’) with a total YP-CORE score ranging from 0 to 40. The YP-CORE was chosen because it is a clinically relevant measure to assess changes in psychological distress in the age group being studied in the current trial, and because it has demonstrated internal reliability (*α* = 0.80) and test-retest reliability across 1 week (*r* = 76, 95% CI 0.65 to 0.86) [34]. The YP-CORE is administered at assessment/baseline, mid-point/6 weeks, endpoint/12 weeks, and follow-up/24 weeks.

##### Client Service and Receipt Inventory (CSRI)

The primary measure for evaluating cost-effectiveness is the CSRI, tailored towards the trial population. The CSRI is a measure widely used to comprehensively record support and services received by research participants (e.g. [43–46]). In the current trial, the CSRI collects information on service utilisation, school support, and accommodation in a manner commensurate with estimating costs [47]. The CSRI is administered at assessment/baseline and follow-up/24 weeks.

#### Secondary outcomes

A range of secondary, self-report outcome measures is collected in both groups. At assessment/baseline, mid-point/6 weeks, endpoint/12 weeks, and follow-up/24 weeks the SDQ [35] is collected to measure psychological difficulties, the Revised Children’s Anxiety and Depression Scale–Short Version (RCADS-SV) [48] collects data on depression and anxiety, the Rosenberg Self-esteem Scale (RSES) [49] collects data on self-esteem, the Student Engagement Scale–Behavioral Engagement sub-scale (SES-BE) [50] collects data about behavioural engagement at school, the Warwick-Edinburgh Mental Well-being Scale (WEMWBS) [51] collects data about level of well-being, and the Goal Based Outcome Record Sheet (GBORS) [52] measures degree of attainment towards personal goals. In addition, at endpoint/12 weeks, the Experience of Service Questionnaire (CHI-ESQ) [53] is administered to measure satisfaction with treatment provision. At the start of each counselling session, participants in the SBHC group are asked to complete the Outcomes Rating Scale (ORS) which assesses areas of life functioning known to change as a result of a therapeutic intervention [39].

In addition, at assessment/baseline and the follow-up/24-week point, schools provide data for educational indicators for each participant in the trial. This will include attendance rate, exclusion rate, detentions and disciplinary proceedings over the previous 3 months as well as current grades.

#### Mediators/predictors

All participants complete a demographic questionnaire at assessment/baseline. Additionally, in order to evaluate whether changes in young people’s levels of psychological distress is mediated by the quality of the relationship that they have with a professional (either counsellors or pastoral care teachers), and/or the quality of the alliance that they experience with their counsellor (for those allocated to SBHC), at mid-point/6 weeks we administer mediating/process measures of ‘Rogerian’ conditions (using the Barrett-Lennard Relationship Inventory Student Form (BLRI OS-40 T-S), [54] in both groups; and the therapeutic alliance (using the WAI-S [42]) in the SBHC group.

Counsellors and supervisors complete demographic questionnaires developed for the study. A researcher completes a School Characteristics Form, developed for the study, with a member of the school’s pastoral care team. This is to obtain data on the size and type of school, pastoral care provision, and characteristics of the general school population.

In the SBHC group, in the first counselling session, the counsellor completes the Current View [55] to record the young person’s characteristics and their presenting issues.

Adherence to the SBHC model is also measured (see above).

#### Qualitative interviews

Qualitative interviews are conducted with a sub-sample of participants, school staff and parents/carers at endpoint/12 weeks. Interviews seek to explore the schools’ staff perceptions on the effect of SBHC on the wider school, parents’/carers’ perceptions of the effect of SBHC on their children, and participants’ experiences of SBHC. We aim to conduct interviews with 50 participants from the SBHC group, and 20 parents/carers. In addition, ten focus groups with school staff will be conducted. The sample for the qualitative analysis is purposively sampled, to include ten schools out of the 18 schools participating in the research. The sampling strategy aims to ensure a mixture of local-authority-maintained schools, and academy schools; as well as a mixture of schools that have been awarded ratings of ‘needing improvement’, ‘satisfactory’, ‘good’, or ‘outstanding’ by the UK Office for Standards in Education. The sampling strategy also aims to ensure that the sub-sample of participants, school staff and parents/carers are drawn from schools of different sizes (in terms of the total population of pupils); and a mixture of same-sex and mixed-sex schools.

Once a school has been selected, all participants in the most recent cohort are asked to take part in a qualitative interview. This includes participants who have chosen to not take up all ten sessions of SBHC offered. In these cases, participants are not interviewed until the end of the intervention period for their cohort. Interviews are between 45 min and 1 h, and are scheduled during the school day. Questions focus upon participants’ views and experiences of SBHC and include questions about how they felt about being offered SBHC, their views about the counsellor, and what was particularly helpful and unhelpful about SBHC.

Key pastoral care staff in selected schools will be interviewed. In some settings it is possible to organise focus groups with relevant pastoral staff, while in others it is more appropriate and practical to organise one-to-one interviews with the main ETHOS school contact. Interviews last for approximately 1 h and questions focus upon understanding any changes that school staff have observed in the participants in the SBHC group, as well as any school-level change.

In addition, we approach all parents of participants in the most recent SBHC cohort. Interviews are conducted over the telephone and last between 30 to 40 min and aim to understand the impact of SBHC on the young person from the parent/carer perspective.

A thematic analysis will be conducted using NVivo for all interview data [56]. This method of analysis is used to examine and explore patterns or themes in the data. The following phases of analysis will be followed: (1) researchers familiarise themselves with the data by reading transcripts of interviews; (2) a list of initial codes across all interviews is generated; (3) potential themes are searched for by collating codes; (4) researchers review the themes in relation to the coded extractions; and (5) themes are refined.

The analysis of participant interviews will be designed to evaluate and refine an a priori logic model which draws on previous evidence to propose a theoretical framework for how SBHC engenders change [31].

#### Adherence

In order to assess whether changes in SBHC participants’ level of psychological distress are associated with counsellors’ adherence to SBHC competences, we will obtain independent adherence ratings via the aforementioned method, and use these to assess the relationship between adherence and outcome.

#### Pastoral care log

A member of the pastoral care team records any instances of care as usual delivered to participants in the trial in a Pastoral Care Log throughout the trial. This data will be used to assess the level and nature of pastoral care delivery across included schools, and any mediating impact on outcomes.

#### Adverse event monitoring

Individuals in contact with trial participants (including pastoral care staff, assessors, counsellors (and their supervisors) testers and qualitative interviewers) are required to use an Adverse Events (AEs) Reporting Log to record the occurrence of any AE in a trial participant throughout the trial (see below).

### Sample size

Sample size was calculated to take into account clustering and participants lost to follow-up. Firstly, without either clustering or participants lost to follow-up, for 90% power to detect a standardised mean difference (SMD) of 0.5, 86 participants would be required per arm (172 in total). The effect size was determined by pooling findings on the primary outcome from four previous studies and making a conservative estimate [24, 31–33]. Intra-class correlation coefficient (ICC) for counsellors was estimated from prior data as 0.05 [24, 31–33]. On average, we estimated that nine young people could be seen per counsellor (4.5 on average per assessment phase); if there is 20% loss to follow-up, this leaves a mean of 7.2. The ICC and average number of young people together leads to a design effect of 1.31, which, when multiplied by the pre-cluster sample size, gives 1.31 × 86 = 113 (rounded up). Hence, after loss to follow-up has taken place, 113/7.2 = 16 (rounded up) practitioners are required per arm. Add 1 [57] to give 17 counsellors. To find the number before loss to follow-up, we calculated 17 × 9 = 153 participants required per arm and 153 × 2 = 306 in total.

### Data quality and management

#### Training of assessors and testers

All assessors and testers are provided with comprehensive training in the study and the purposes and principles of the research meetings, including key study documentation, before conducting any research meetings with participants. Assessors have a diploma in counselling or psychotherapy (or are currently enrolled in a diploma-level training in counselling or psychotherapy or equivalent) and have experience of working with young people (aged 13 years onwards) and using outcomes measures in clinical assessment. Study-specific assessment guidelines have been developed for the trial and are used in assessment training, and each assessment meeting. These provide guidance on administration of all measures at baseline, and evaluating eligibility of each potential participant. This includes risk assessment and management, and assessment of Gillick competency. Testers have a completed (or are currently studying for) a postgraduate course in psychology, counselling, or a related discipline. The well-being of the young person is of paramount importance and testers are trained to sensitively work with significant levels of distress if it arises during a research meeting. Study-specific tester guidelines have been developed for the trial and are used in tester training, and each testing meeting. These provide guidance on administration of all measures at 6 weeks/mid-point, 12 weeks/endpoint and 24 weeks/follow-up. The assessor and tester guidelines contain a glossary of words/phrases used in the outcome measures that participants may struggle to comprehend. Assessors and testers are trained to use this glossary, as needed, to ensure that the process of administrating measures is as standardised as possible.

#### Data entry and storage

All personal data (e.g. signed consent forms, participants’ date of birth) are stored in a locked cabinet. Anonymised quantitative measures and educational attainment/engagement data are collected on hard copies and stored in a separate locked filing cabinet from personal data. Partially anonymised data (e.g. audio-recordings of sessions) are kept temporarily on password-protected, encrypted, recording Android devices, and transferred to encrypted, password-protected servers at the University of Roehampton. Anonymised quantitative measures are password protected and submitted as electronic copies to MAHSC-CTU. Scanned copies of CRFs transferred to the CTU are stored securely within MAHSC-CTU offices and in accordance with the Data Protection Act [58]. The trial adheres to the Economic and Social Research Council’s (ESRC) Research Data Policy and the Centre for Research in Social and Psychological Transformation (CREST, Department of Psychology at the University of Roehampton), Data Storage and Protection Procedures.

Data will be entered by MAHCS-CTU staff onto DBS: a restricted-access and bespoke-validated clinical trial database managed by MAHSC-CTU. Data validation is run on the data entered and data queries and corrections are sent to the researchers to clarify and correct anomalous data. Prior to the end of the study, quality control checks will be conducted, prior to the database being finalised.

#### Trial monitoring

Trial monitoring will be performed by the University of Roehampton which will conduct regular audits and site visits to schools; and MAHSC-CTU which will conduct a mixture of remote monitoring of essential documentation and on-site monitoring of source data. The site checks will aim to verify that the rights and well-being of participants are protected; verify accuracy, completion and validity of reported trial data from the source documents; and evaluate the conduct of the trial within the school with regard to compliance with the approved protocols.

### Confidentiality

Names of participants on the consent forms (personal data) are stored separately from anonymised data in locked filing cabinets and only accessible by named personnel. A separate code (‘pre-randomisation code’) links names to Participant ID numbers used in data files, which are password protected and only accessible by named personnel.

### Statistical methods

Statistical analysis will be carried out by the ETHOS principal statistician and economist according to a statistical analysis plan agreed in advance. The principal statistician will be blind to the randomisation, with the exception of analysis of the WAI-S which is only completed by participants in the SBHC group. Allocation for all other measures will be coded as a non-identifiable variable in the clinical effectiveness analysis to minimise potential bias. The principal economist cannot be blind to allocation due to the nature of the data being analysed (e.g. number of counselling sessions attended).

#### Analysis of clinical effectiveness

The primary analysis will be based on the intention-to-treat principle. Per-protocol analysis will also be conducted. Baseline characteristics will be described by group and pooled using mean, standard deviation (SD), minimum, maximum, median, and interquartile range. Categorical variables will be described by frequency. Analyses will be performed using linear mixed-effects models (LMMs) including data from all randomised participants by intention to treat. All variables used for stratification/minimisation will be included as covariates (random intercept for school and practitioner; fixed effect for other baseline characteristics). Differences between SBHC and PCAU will be summarised by 95% CIs of the difference between groups, estimated by the LMMs. Given the likely non-linear patterns of change and measures taken at discrete time points, analyses will be conducted at each time point, adjusted by baseline scores. Standardised mean differences will be calculated using the LMM-estimated mean differences between groups at each time point after baseline, divided by the baseline SD. Sensitivity analyses will be conducted to test for any moderating effects of support provided to participants to complete measures. Participants with and without missing data will be compared using baseline characteristics, including intervention allocation, to test for patterns of missing values and systematic biases. Multivariate imputation by chained equations will be used to compute questionnaire scores for participants with missing item responses.

Secondary analysis will include modelling of the extent to which the relationship between intervention allocation and outcome is mediated by the BLRI variables, and testing for differences in the frequency of AEs by allocation. Data will also be analysed within the treatment group to determine whether outcome is predicted by mid-point therapeutic alliance, while adjusting for all baseline variables.

#### Cost-effectiveness analysis

Costs and cost-effectiveness will be assessed for the follow-up point (24 weeks) using a standard economic analysis framework, shown to be appropriate in an earlier trial [59]. A unit cost will be sought for each service used, including SBHC, by young people in the ETHOS trial and recorded on the database (e.g. [47, 59, 60]. To facilitate estimation of the full costs of SBHC, additional data will be collected from SBHC counsellors (for example, on time spent travelling, liaising with professionals, or in supervision) and from those organising the counsellor service. The amount of each service used by each young person will be multiplied by the appropriate unit cost and summed to arrive at a total support cost per young person for the baseline and follow-up (24 weeks) time points.

Descriptive statistics (frequencies, percentages, means, range, SD) will be used to compare the support packages and costs at each time point for each group. Service and support costs will be identified by funder, including those supports and services provided and funded by the school. Care will be taken to identify any systematic cost differences between locations (schools) as the local array of services may differ, leading to variations in access and, therefore, use. Cost and outcome data will be compared over time and between the SBHC and PCAU group and incremental cost-effectiveness ratios (ICERs) calculated using the change in primary outcome (YP-CORE). In the event of the SBHC group having both higher costs and generating improved outcomes, the current approach is to estimate cost-effectiveness acceptability curves (CEACs). The net monetary benefit for the outcome measures will be calculated and the proportion of bootstrapped estimates of the group difference favouring SBHC will be plotted with corresponding values of willingness to pay.

### Risk procedures and reporting of adverse events (AEs)

Written protocols for managing risk, and monitoring and reporting AEs and serious adverse events (SAEs) is followed for all trial participants throughout the trial (from the point of enrolment into the trial, to the follow-up/24-week time point). An adverse event (AE) is defined as any negative psychological, emotional or behavioural occurrence, or sustained deterioration in a research participant. In the current trial, we have included arrest by police; running away from home; excluded from family home; school exclusion; significant decrease in school attendance; significant deterioration in behaviour, including threatening violence, exhibiting violent behaviour or serious injury to another person, exposure to violence or abuse; significant increase in emotional difficulties; self-harm (if not a presenting issue), or escalating self-harm (when it is a presenting issue); a complaint made against the counsellor, or an issue with the counsellor, resulting in discontinuation of counselling; suicidal ideation; suicidal intent; hospitalisation due to drugs or alcohol, or for psychiatric reasons; and death, including suicide.

An Adverse Events Reporting Form is used by all individuals in contact with participants, who are trained to recognise and respond, in an ethical and timely way, to risk and any issues relating to safeguarding. The overall safety of participants is the responsibility of the trial’s chief investigator (CI). However, in practice the CI relies on all research staff, counsellors, supervisors and school staff to ensure that AEs are identified and addressed in an appropriate and timely manner. Thus, safety is a shared responsibility. Individuals completing the form are asked to consider whether the AE is serious, defined as any AE which is life threatening or results in death, and whether it may be a result of participating in the trial. The severity of each AE is also assessed, according to its intensity, duration and the degree of impairment to the young person (or, when relevant, another person such as in case of risk to others). Severity is graded as ‘mild’, ‘moderate’, ‘severe’, ‘very severe’, or ‘extremely severe’.

The CI has responsibility for reviewing and signing the AE Reporting Forms, for ensuring that the relevant school staff member is aware of the occurrence of any AEs or SAEs at their school; and the ETHOS clinical lead (CL) in instances where the AE or SAE has occurred in a participant in the SBHC group. The CI is also responsible for reporting the AE or SAE to the Chair of the Data Monitoring and Ethics Committee (DMEC) (see ‘Governance and oversight’ below). In the case of SAEs, or those deemed related to participating in the trial, expedited reporting procedures are followed, which includes a reporting timeframe of one week from receipt of the AE Reporting Form.

### Public involvement

A panel of young people (drawn from the Young Person’s Advisory Group at the National Children’s Bureau, NCB) and a panel of parents and carers (drawn from the Parent and Carers Advisory Group at NCB) has advised on the development of an appropriate engagement strategy to keep young people (and parents and carers where appropriate) abreast of project developments. The aim is to ensure that the trial explores issues of relevance to young people, and minimises participant attrition. Representatives from both panels have been invited to join the Trial Steering Committee (see below). Panel members include young people who have received training from the NCB Research Centre in a range of research skills (e.g. appraising the academic literature, reviewing engagement materials, co-producing research summaries). We have aimed to involve these panel members at all stages of the study where possible, and look to include them in our dissemination activities where we know that peer feedback is a highly effective way of ensuring that research findings reach the intended audience.

### Governance and oversight

A Trial Steering Committee (TSC) has been established with an independent chair, and representatives from different, relevant professional groups (including a counselling academic, an educationalist, a representative from the ESRC (funder), an independent statistician, and an independent economist), a representative young person and a representative parent/carer, together with members of the research team. The role of the TSC is to monitor the scientific integrity of the trial, the scientific validity of the trial protocol, assessment of the trial quality and conduct as well as for the scientific quality of the final trial report. Decisions about the continuation or termination of the trial or substantial amendments to the protocol are the responsibility of the TSC.

A Data Monitoring and Ethics Committee (DMEC) has also been convened under the direction of an independent chair. The DMEC comprises a clinician, a separate statistician from the TSC, who is also independent of the Trial Management Group (TMG) (see below) and similarly, a separate and independent economist. The role of the DMEC is to review accruing trial data and to assess whether there are any safety issues that should be brought to the participants’ attention, whether any safety amendments should be made or if there are any reasons that the trial should not continue. Open reports are provided to the DMEC by the TMG and closed reports are provided by the CTU. The DMEC Chair reports the DMEC’s recommendations to the Chair of the TSC and may request additional reports or information if required.

A TMG has been established and includes those individuals responsible for the day-to-day management of the trial including the CI, project manager, principal statistician and economist, and all co-researchers. Notwithstanding the legal obligations of the lead organisation (University of Roehampton) and the CI, the TMG has operational responsibility for the conduct of the trial including monitoring overall progress to ensure that the protocol is adhered to, and taking appropriate action to safeguard the participants and the quality of the trial if necessary.

## Discussion

The ETHOS study is the first RCT powered to detect clinically meaningful differences investigating the clinical and cost-effectiveness of SBHC, compared to PCAU. Evidence of the effectiveness of psychotherapeutic interventions with children and adolescents comes mainly from trials of CBT for the treatment of anxiety and depression. As many young people referred to school counselling services are more likely to be experiencing emotional distress as a result of a range of life difficulties, rather than a specific clinical disorder, there is a need for school-based interventions that address these needs. SBHC presents one such potential intervention, and the results from four pilot trials provide preliminary evidence of clinical effectiveness. Determining the clinical and cost-effectiveness of SBHC is important for all stakeholders, including policy-makers, statutory advisory bodies for child welfare, head teachers, children and young people practitioners, child welfare and parenting organisations, and young people.

Conducting the current trial in a school (real-world) setting is a pragmatic approach to assessing the effectiveness of SBHC compared to PCAU, with the added advantage of being able to evaluate the intervention in a way that mirrors routine practice. School pastoral care staff are particularly well placed to identify potentially eligible participants, and so working with schools during the pre-screening period may also be beneficial to the trial’s recruitment rates. A further advantage of conducting the current trial in a school setting pertains to school culture and day-to-day structures (e.g. the use of regular timetables, monitoring of pupil attendance), which lends itself well to scheduling and completing data collection at our four time points.

There are also significant challenges to conducting the current trial within a school context. Necessary protocols for ethical research practice can present a burden to included schools, which are not familiar with the research-related administration involved in running a trial of this size. Equally, the trial’s schedule of time points for data collection and counselling sessions needs to take into account the various academic calendars across schools, including such things as school holidays, site inspections carried out by the Office for Standards in Education, and in-service, school training days for staff. These particular challenges have necessitated training provision and regular debriefing in the research aspects of the trial, with schools; and the nature and realities of school life to our researchers, as well as developing and maintaining strong working relationship between sub-teams. A final challenge of conducting ETHOS in a school environment pertains to the heterogeneous nature of our schools’ pastoral care provision, which may present particular challenges at the point of analysis. We aim to address this challenge through collecting data on each school’s pastoral care provision in the School Characteristic’s Form developed for the study, and by collecting information relevant to each participant’s engagement with their school’s pastoral care services regarding type, regularity and length of time, in a Pastoral Care Log also developed for the study.

In addition, a considerable challenge has included developing a protocol for monitoring and reporting AEs in counselling. The academic literature regarding AE monitoring in RCTs generally relates to trials of pharmacological interventions and there is scant academic literature in the counselling and psychotherapy fields. This has require us to utilise a process of adopting and adapting a model of monitoring and reporting more commonly applied in pharmacological studies, as well as drawing on the clinical expertise within the core research team to inform protocol development. We view our current approach as iterative, and aim towards being able to share an established, more definitive, set of protocols with the counselling research community on trial completion.

A further challenge includes obtaining parent/carer consent prior to the assessment meeting with the young person. While our method of ‘opt-in’ consent was ethically necessary, it can be time consuming to obtain and has presented school staff with additional administrative duties. This has been largely overcome by working closely with school staff as early as possible in the process of identifying young people for the project and engaging their parent/carer. However, this method may also be contributing to the variable recruitment rates we have observed across included schools, as it has required parents and carers to be sufficiently engaged with their child’s school, as well as being able to read English at a sufficient level to understand the Information Sheet for Parents/Carers. Parent/carer ‘opt-in’ consent represents a potential source of selection bias, as we are only including participants who have been willing to involve their parent/carer in their decision to take part in the trial and whose parent/carer has agreed to their involvement; and in terms of only including participants and parents/carers with a sufficient level of English language comprehension.

The results of this trial will contribute significantly to the evidence base for SBHC and to the wider field of adolescent mental health interventions. Our data also has the potential to inform the development of national guidelines for mental health support for schools and make a direct contribution at a policy level, by providing up-to-date and reliable information about the utility of school counselling. A trial that is powered to detect clinically meaningful differences also provides an opportunity to develop an understanding of the process of change in SBHC, and to trial the newly established competency framework for humanistic counselling with children and young people [30]. Furthermore, the research will be used to develop and test a manual for the effective implementation of SBHC by counsellors and psychotherapists.

### Trial status

ISRCTN Registry, ISRCTN10460622 (11 May 2016). The study commenced recruitment in September 2016 and recruitment due for completion in February 2018.

## Additional file

Additional file 1: SPIRIT 2013 Checklist: recommended items to address in a clinical trial protocol and related documents. (DOC 122 kb)

## Abbreviations

AE: Adverse event
BACP: British Association for Counselling and Psychotherapy
BLRI: Barrett-Lennard Relationship Inventory
CBT: Cognitive behaviour therapy
CHI-ESQ: Experience of Service Questionnaire
CI: Confidence interval
CL: Clinical lead
CREST: Centre for Research in Social and Psychological Transformation
CRF: Case Report Form
CSRI: Client Service Receipt Inventory
CYP PRN: Children and Young People Practise Research Network
DMEC: Data Monitoring and Ethics Committee
ES: Emotional Symptoms sub-scale of the SDQ
ESRC: Economic and Social Research Council
ETHOS: Effectiveness and cost-effectiveness trial of humanistic counselling in schools
GBORS: Goal Based Outcome Record Sheet
ICC: Intra-class correlation coefficient
ICER: Incremental cost-effectiveness ratio
ISRCTN: International Standard Randomised Controlled Trial Number
LA: Local Authority
LMM: Linear mixed-effects models
MAHSC-CTU: Manchester Academic Health Science Centre, Trials Coordination Unit
NCB: National Children’s Bureau
NICE: National Institute for Health and Care Excellence
ORS: Outcome Rating Scale
PCAU: Pastoral Care as Usual
PCEPS-YP: Person Centred and Experiential Psychotherapy Scale – Young People
RCADS-SV: Revised Child Anxiety and Depression Scale–Short Version
RCT: Randomised controlled trial
RSES: Rosenberg Self-Esteem Scale
SAE: Serious adverse event
SBHC: School-based humanistic counselling
SD: Standard deviation
SDQ: Strengths and Difficulties Questionnaire
SES: Student Engagement Scale
SMD: Standard mean difference
SPIRIT: Standard Protocol Items: Recommendations for Interventional Trials
SQL: Standard Query Language
TMG: Trial Management Group
TSC: Trial Steering Committee
UK: United Kingdom
UNICEF: United Nations International Children’s Emergency Fund
WAI-S: Working Alliance Inventory – Short Form
WEMWBS: Short Warwick-Edinburgh Mental Well-being Scale
WHO: World Health Organisation
YP-CORE: Young Person’s Clinical Outcomes in Routine Evaluation
